# Paired Electrosynthesis at Interdigitated Microband Array Electrodes without Intentionally Added Electrolyte: C–C Coupling of Dicyanobenzenes with Methanol

**DOI:** 10.1021/acs.jpcc.4c07899

**Published:** 2025-02-03

**Authors:** Tingran Liu, Claire McMullin, James E. Taylor, Frank Marken

**Affiliations:** Department of Chemistry, University of Bath, Claverton Down, Bath BA2 7AY, U.K.

## Abstract

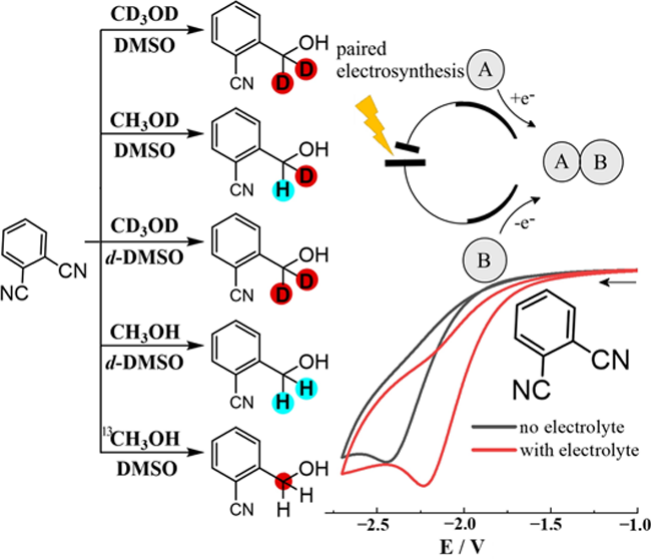

The formation of substituted benzyl alcohols from dicyanobenzenes and methanol (C–C coupling) is demonstrated as a model system for paired electrosynthesis and investigated at interdigitated microband array electrodes in a microreactor with/without intentionally added supporting electrolyte. A Pt–Pt microband array with 5 μm bands separated by 5 μm gaps is employed in a dimethyl sulfoxide (DMSO) solvent. Yields are optimized to approximately 50% at the point of 100% conversion. The mechanism is investigated by employing isotope labeling (CD_3_OD, CH_3_OD, *d*_6_-DMSO, ^13^CH_3_OH). The methylene group (^12^C or ^13^C) is obtained with H_2_, D_2_, and DH substitution patterns, and a hypothesis for a corresponding mechanism is discussed aided with density functional theory (DFT) calculations. Implications for sustainable electrosynthesis at paired microband electrodes are discussed.

## 1 Introduction

Electrosynthesis utilizing electricity to trigger molecular transformation near/at the electrode surface^1,2^ attracted the attention of researchers ever since the discovery of Kolbe’s carboxylate decarboxylation reaction in 1849.^3^ Strategies for electrosyntheses were proposed on lab scale as well as on industrial scale.^4−6^ There are still many challenges including complexity in mechanisms and a requirement for innovation in reactor design.^7^ In 1994, interdigitated array electrodes were introduced to electrosynthesis by Girault and co-workers for cases such as propylene epoxidation or methoxylation of furan at interdigitated platinum electrodes.^8,9^ Interdigitated microband electrodes can be beneficial due to low Ohmic loss across anode and cathode (for very small interelectrode gaps). To improve sustainability and to avoid separation steps, processes at interdigitated microbands can be performed in the limit of no added electrolyte.^10^ Importantly, anodic and cathodic processes occur within micrometers (within the diffusion zone) of each other and are therefore paired.^11,12^ Intermediates (even short-lived; for a diffusion layer δ = 5 μm, the transit time is typically τ = δ^2^/2*D* ≈ 12 ms) from the anode and cathode can interact.

**Figure 1:**
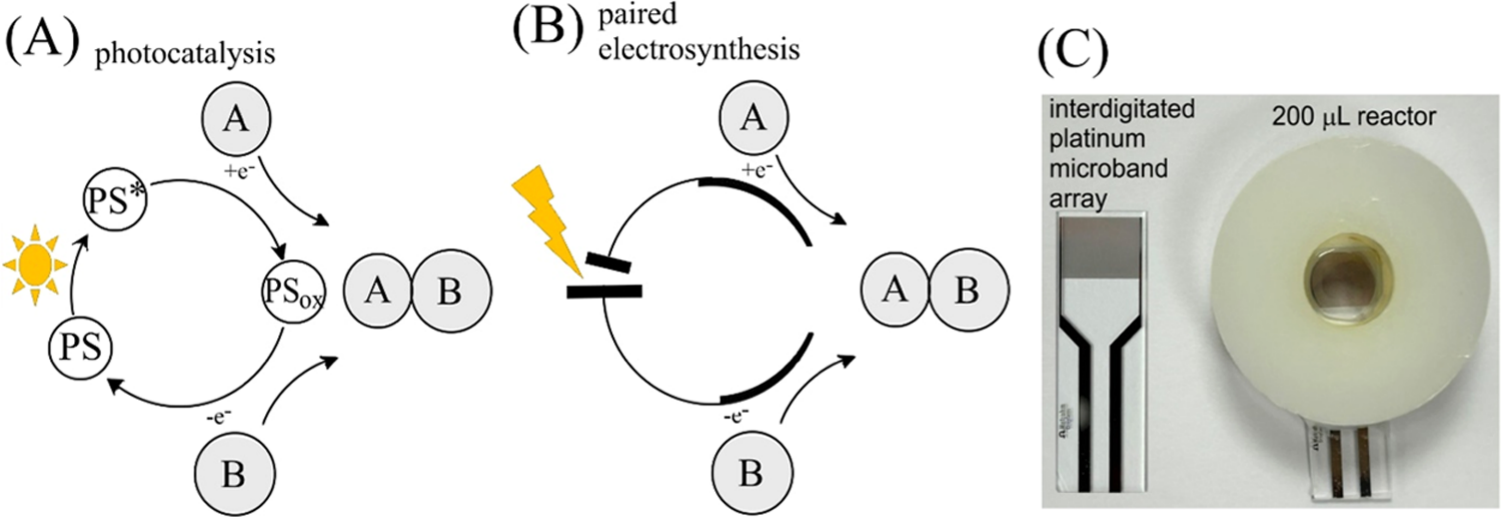
Schematic for (A) a photocatalytic process based on two single-electron transfers and (B) a paired electrochemical process mimicking the conditions by placing the anode and cathode very closely together (diffusion distance). (C) Image of the interdigitated microband electrode array and the electrosynthesis reactor.^10^

Benzonitriles are commonly used precursors in pharmaceutical synthesis. The nitrile group is a functional group that can be reduced or transformed or replaced into a new functionality. Nitrile is a good leaving group that upon electron transfer provides a radical building block, for example, under photocatalysis conditions.^13^ Electrosynthesis based on sustainable electricity^14^ has been proposed/employed in conjunction with nitriles to generate reactive intermediates. Jensen and co-workers proposed a strategy that makes the single-electron transfer (SET) process possible *via* electrochemistry by using interdigitated microband electrodes under flow cell conditions.^15,16^ It was realized that there is a possibility to “translate” processes in photocatalysis (based on multiple SETs involving a sensitizer dye) to paired electrosynthesis (based on potential controlled electron transfer at closely spaced anode and cathode). Figure 1A,B describes the general scheme for “translating” a photocatalytic reaction into an electrochemically driven paired electrosynthesis. Figure 1C shows an interdigitated platinum microband array electrode with a polymer washer to give a 200 μL electrosynthesis microreactor.^5^ The reaction space is defined by the microband geometry (diffusion–migration) and convection (to supply reactants in irreversible electrode reactions). Interdigitated microband arrays could be attached to reactor walls or even to stirrers to operate on conventional reaction ware. Here, the microreactor scale without convection is sufficient for exploratory work on the mechanism and optimization of paired electrosynthetic processes.

Previously, electrosynthesis without intentionally added electrolyte self-supported^17^ has been successfully applied under flow cell conditions, with microgap parallel-plate electrodes,^18,19^ in fuel cell-type systems,^20^ or using amine-based electrolytes instead of salts^21^ to eliminate the separation step to obtain the product. For a sufficiently narrow gap between the microband electrodes, interdigitated electrode (IDE) structures could potentially be used without any added supporting electrolyte.

**Scheme 1:**
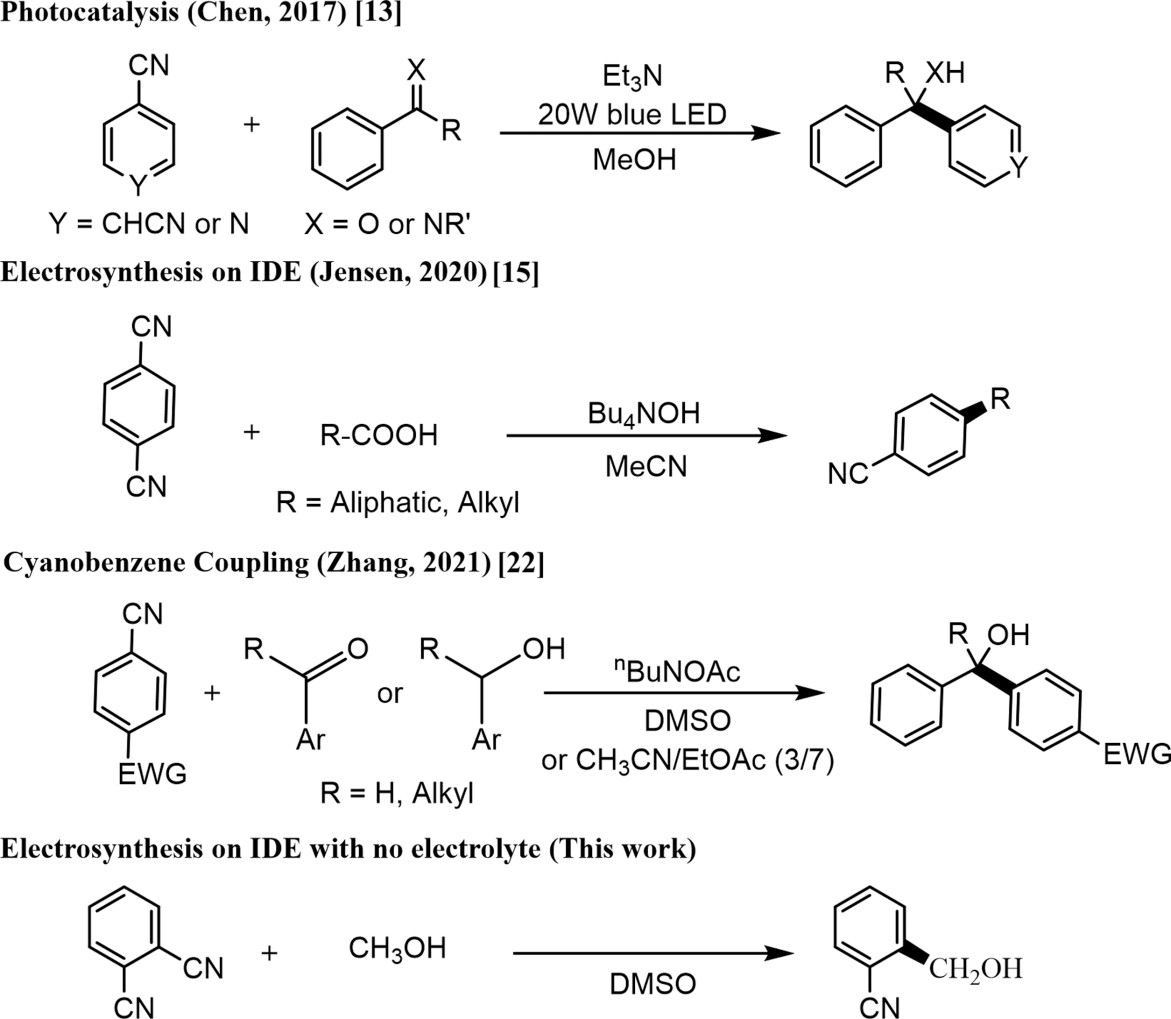
Scheme for Benzonitrile Photoreduction (Chen, 2017),^13^ Paired Electrosynthesis with Carboxylates (Jensen, 2020),^15^ Paired Electrosynthesis with Aldehydes (Zhang, 2021),^22^ and Paired Electrosynthesis with Methanol and without Added Supporting Electrolyte

Herein, a supporting electrolyte-free C–C coupling in dimethyl sulfoxide (DMSO) is employed in an interdigitated microband electrode array cell. Similar types of reactivity have been reported (Scheme 1) for photochemical and electrochemical processes. Here, an interdigitated microband array electrode is employed with a 5 μm gap/5 μm width attached washer and forms a cell (200 μL volume) for electrosynthesis at the NMR scale (Figure 1C). DMSO is the solvent of choice due to a low rate of evaporation and due to high polarity to help maintain conductivity in electrolyte salt-free conditions during electrolysis. Important here is also the high solubility of the starting material in DMSO. By applying potential, dicyanobenzene reduction coupled to methanol oxidation leads to C–C coupling products. Isomers of dicyanobenzene are investigated and isotope effects in the electrosynthesis are employed to trace reaction pathways and intermediates.

## 2 Experimental Section

### 2.1 Reagents

Dimethyl sulfoxide (DMSO) and methanol (MeOH) were purchased from Sigma-Aldrich and utilized without further purification. Regents such as tetrabutylammonium perchlorate (TBAP), 1,2-dicyanobenzene, 1,3-dicyanobenzene, and 1,4-dicyanobenzene were purchased from Sigma-Aldrich. Ethyl acetate (EtOAc) and *n*-hexane for purification steps were purchased from Sigma-Aldrich. Isotope reagents *d*-CDCl_3_, CH_3_OD, CD_3_OD, and ^13^CH_3_OH were purchased from Sigma-Aldrich.

### 2.2 Instrumentation

A platinum disk electrode (1.6 mm diameter) was purchased from Alvatek (U.K.). Platinum interdigitated microband electrode arrays (5 μm bands, 5 μm gap, glass substrate, array of 250 anodes and 250 cathodes, active area 5 mm × 6 mm, total area as width × length of bands = 0.3 cm^2^, G-IDEPT5, Metrohm) were purchased. Galvanostatic or potentiostatic electrolyses were performed with a Micro-Autolab II (Metrohm, U.K.) system with computer control. NMR spectra were obtained with a 400 MHz Bruker Neo spectrometer equipped with an iProbe. Chromatography was conducted with a CombiFlash NextGen 300^+^ system from a TELENYNE ISCO.

### 2.3 Electrosynthesis Procedure

(A) Microreactor scale. Interdigitated microband electrodes on glass were attached to a plastic washer by a silicone sealant (ACC Silicones, Silicoset 15) as the reactor cell. Syntheses were performed with typically 0.2 μL of 0.2 M dicyanobenzene in DMSO as the solvent with 25 vol % methanol added. After 12 h of electrolysis, the liquid was transferred into a nuclear magnetic resonance (NMR) tube with 10% of CDCl_3_ added for NMR analysis. (B) Bulk scale. For larger-scale electrolysis, the cell was used repeatedly (five repeats to give typically 10 mg of product). The reactor contents were combined and purified by flash chromatography (CombiFlash NextGen 300^+^, petroleum ether/EtOAc with EtOAc from 0 to 40%, 13 mL min^–1^, 254 nm) to afford the product. Thin-layer chromatography (TLC) was used to monitor reaction progress (mobile phase 40% EtOAc/PE with a product *R_f_* value of 0.45).

### 2.4 Computational Methodology

Density functional theory (DFT) calculations were performed to determine the relative energies of intermediates with Gaussian 16 (C.01).^23^ All atoms were described with the 6-311++G** basis set.^24,25^ Initial BP86^26,27^ optimizations were performed using the “grid = ultrafine” option, with all stationary points being fully characterized *via* analytical frequency calculations as minima (all positive eigenvalues). All energies were recomputed to correct for the effect of DMSO (ε = 46.826) solvent and dispersion. Single point calculations were run using the polarizable continuum model, with the keyword “scrf = DiMethylSulfoxide”,^28^ and also employing Grimme’s D3 parameter set with Becke-Johnson damping as implemented in Gaussian.^29^

## 3 Results and Discussion

### 3.1 Effect of Supporting Electrolyte: Voltammetry and Electrosynthesis

**Figure 2:**
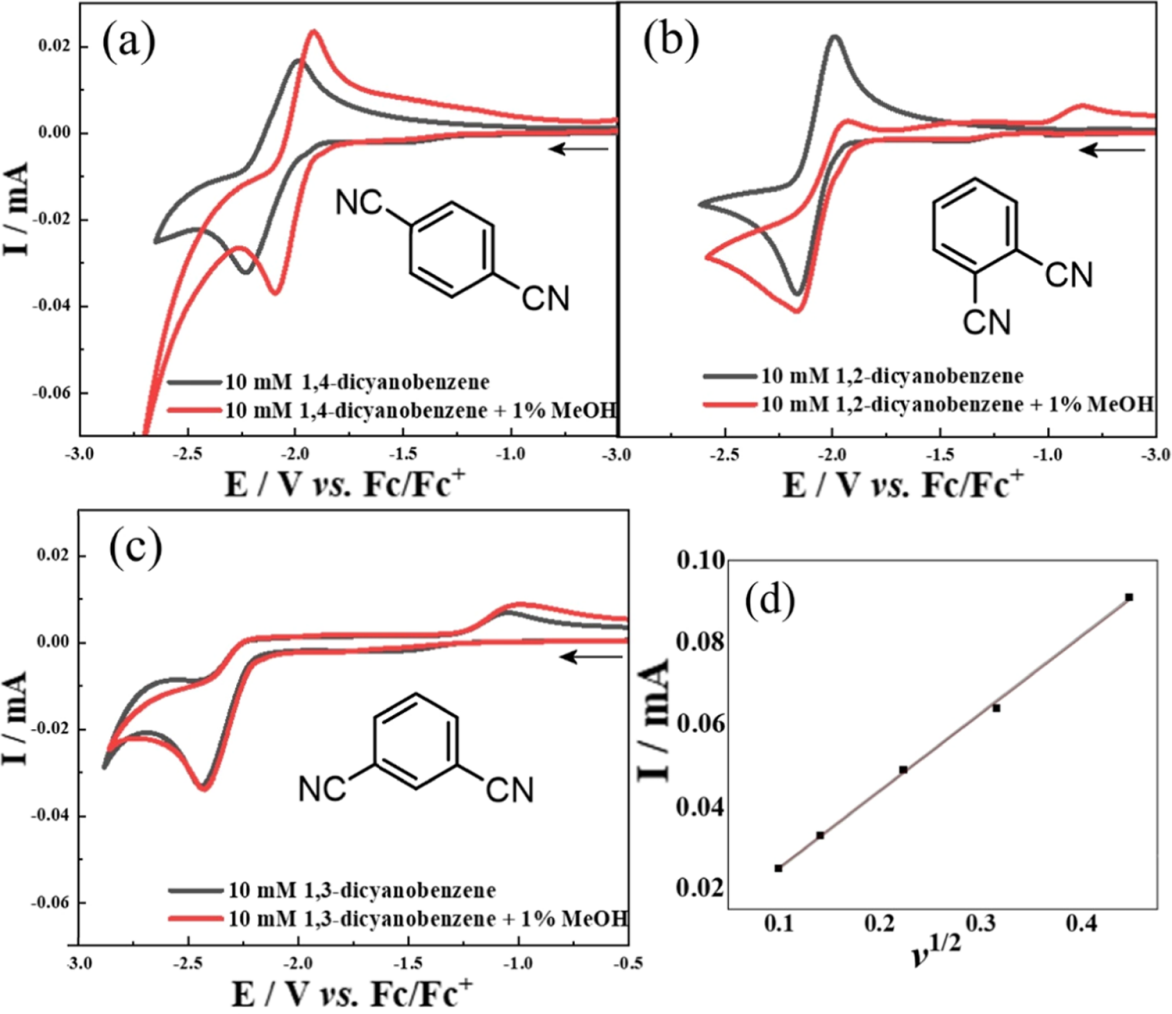
Cyclic voltammograms (1.6 mm diameter Pt disk; scan rate of 20 mV s^–1^) for 15 mM dicyanobenzenes in DMSO (with 0.1 M TBAP electrolyte). A Pt wire counter electrode and a Ag wire reference electrode were employed with ferrocene as internal reference. (a) 1,4-Dicyanobenzene; (b) 1,2-dicyanobenzene; (c) 1,3-dicyanobenzene. Black: pure DMSO/0.1 M TBAP; red: with 1% methanol added. (d) Plot of peak current *versus* √*v*.

First, 1,4-dicyanobenzene, 1,3-dicyanobenzene, and 1,2-dicyanobenzene were investigated electrochemically by employing cyclic voltammetry at a 1.6 mm diameter Pt disk electrode (Figure 2). In the measurement of 1,4-dicyanobenzene and 1,2-dicyanobenzene, a reduction peak was observed (at approximately −2.2 V *versus* Fc/Fc^+^) followed by an oxidation peak upon reversal of the scan direction. For 1,3-dicyanobenzene, the reduction is chemically irreversible due to fast follow-up reactions (benzonitrile is observed as a product). In contrast, the radical anion intermediates for both 1,4- and 1,2-dicyanobenzene appear to be reasonably stable at room temperature in DMSO (over seconds). All peak currents are likely to be associated with one-electron reduction reactions. The effect of methanol (MeOH, 1 vol %) was studied by voltammetry. Red curves in Figure 2 show data for 1 vol % MeOH in DMSO. The one-electron reduction remains reversible for 1,4-dicyanobenzene indicative of slow follow-up chemical steps. The one-electron transfer process for 1,4-dicyanobenzene does shift approximately 0.1 V positive presumably due to the interaction of methanol with the radical anion. For 1,2-dicyanobenzene, the anodic peak is strongly diminished, indicative of a faster reaction step. For 1,3-dicyanobenzene, there is no obvious change in the voltammetric response. For both 1,2-dicyanobenzene and 1,3-dicyanobenzene, the chemically irreversible cathodic process is likely to produce benzonitrile. Only for 1,2-dicyanobenzene, this side reaction can be outrun at the interdigitated microband array electrode (*vide infra*).

In order to determine the diffusion coefficient for dicyanobenzenes in DMSO, the Randles–Sevcik equation (eq 1) was applied. Figure 2d shows an inset with a plot of peak current *versus* the square root of scan rate.
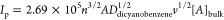
1The *I*_p_*versus* slope is determined as 1.9 × 10^–4^ A (V s^–1^)^−1/2^. The geometrical area of the electrode is *A* = 2 × 10^–6^ m^2^, and the bulk concentration of 1,2-dicyanobenzene is 15 mol m^–3^. The diffusion coefficient is calculated to be typically *D*_dicyanobenzene_ = 0.55 × 10^–9^ m^2^ s^–1^. This value is very close to the corresponding estimated diffusion coefficient based on the equation by Wilke and Chang,^30^ which suggests approximately *D*_dicyanobenzene_ = 0.48 × 10^–9^ m^2^ s^–1^. The processes are clearly one-electron transfer reactions. The diffusion coefficient is useful when studying the current during electrosynthesis.

**Figure 3:**
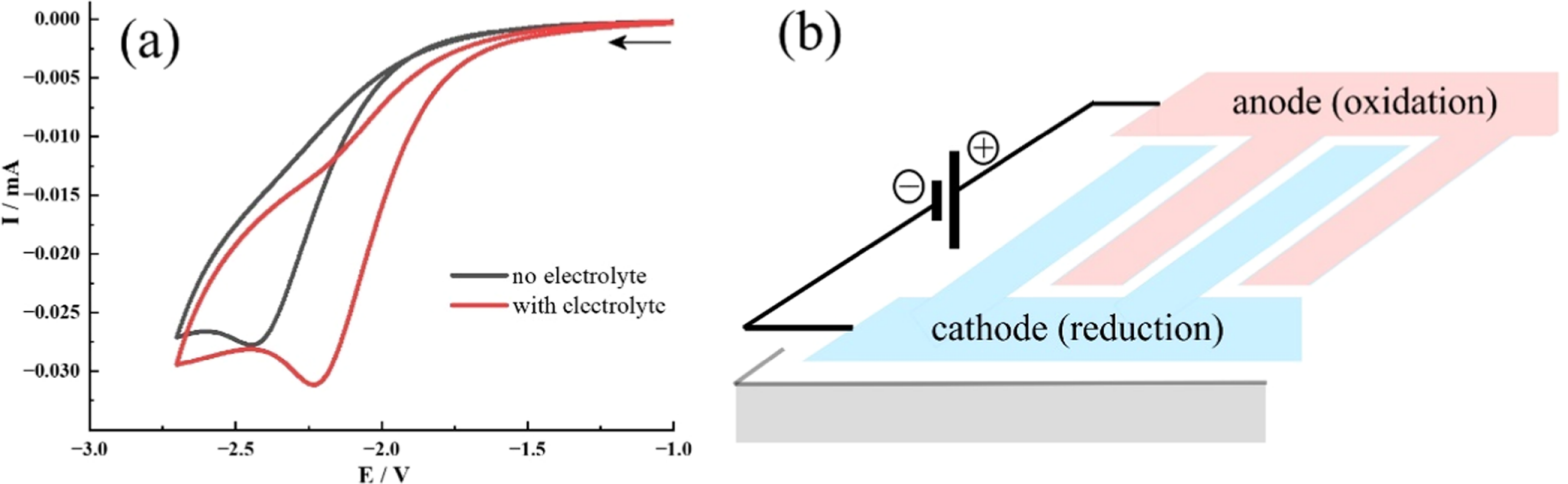
(a) Cyclic voltammograms (Pt interdigitated microband electrode array; scan rate of 20 mV s^–1^; under argon) for 5 mM 1,2-dicyanobenzenes in DMSO (with/without 0.1 M TBAP electrolyte). Black curve: no electrolyte; red curve: with 0.1 M TBAP added. (b) Schematic showing the two-electrode configuration with microband arrays.

Next, the current for 1,2-dicyanobenzene reduction is investigated at the interdigitated microband electrode array. A two-electrode configuration is employed (both the anode and cathode form the interdigitated microband array; see Figure 3b). The comparison of experimental conditions with 0.1 M TBAP added (red curve) and without any intentionally added electrolyte (black curve) suggests only minor changes. The peak current is typically 30 μA for a scan rate of 20 mV s^–1^. Under these conditions feedback (electron shuttling, for example, by radical anion intermediates) between anode and cathode is expected. Note that the anode current and cathode currents are equal (with opposite sign) under these conditions. There is no well-defined steady-state current response probably due to irreversible (nonsteady state due to planar diffusion) anode processes, which are required to start the reduction at the cathode.

An equation for feedback current at interdigitated microband array electrodes derived by Aoki^31^ (here, for the special case of electrode width = interelectrode gap) is given in eq 2.
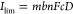
2where *m* = 250 is the number of individual band electrodes (for each anode and cathode), *b* = 6 × 10^–3^ m is approximately the length of bands, *n* = 1 is the number of electrons transferred per molecule diffusing to the electrode, *F* is the Faraday constant, *c* is the bulk concentration, and *D*_dicyanobenzene_ is the diffusion coefficient. The calculated quasi-steady-state current for 5 mM 1,2-dicyanobenezene is *I*_lim_ = 0.4 mA. The observed currents are lower indicative of effects from the anode process. During synthesis using 200 mM 1,2-dicyanobenzene (in the presence of 25% MeOH), the current typically reaches 4 mA at 4 V applied voltage (two-electrode), which is again lower when compared to the anticipated full feedback current *I*_lim_ = 16 mA. Therefore, the irreversibility of the electrochemical process (*i.e*., product formation) under paired electrosynthesis conditions lowers the current although some degree of electron shuttling by feedback will still occur, *vide infra*. That is, the current is producing products rather than cycling only the redox states of the starting material.

**Figure 4:**
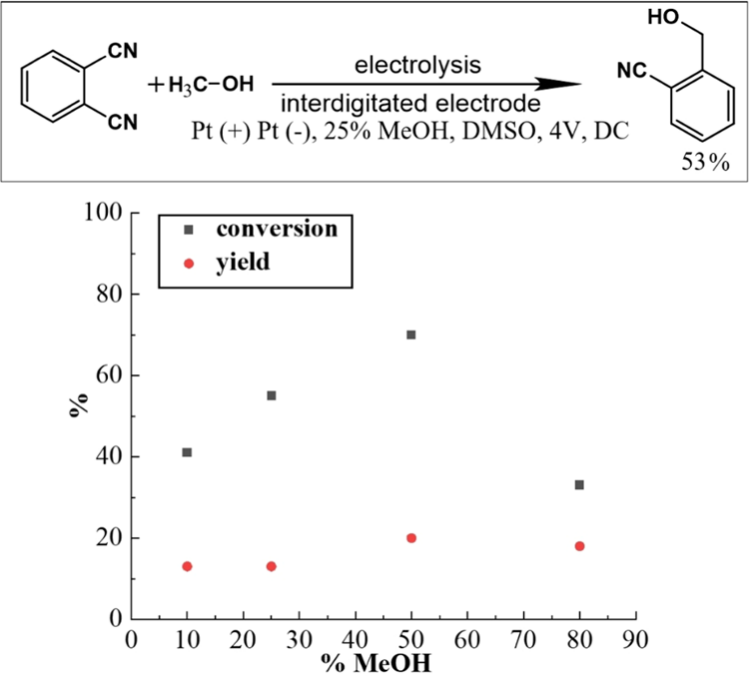
Optimized test conditions for the paired electrosynthesis of substituted benzyl alcohols and plot of MeOH addition (vol %) on the 1,2-dicyanobenzene with MeOH coupling yield (4 V; 12 h); DMSO with 0.2 M 1,2-dicyanobenzene at Pt interdigitated microband array electrode.

When performing the electrosynthesis in a two-electrode configuration, the concentration of methanol is important. The process was performed at 4 V for 4 h and 0.2 M 1,2-dicyanobenzene. The concentration of methanol was varied. Figure 4 shows that both conversion (the loss of starting material) and yield (the formation of product) increase up to about 50 vol % MeOH. More methanol is detrimental. In the following experiments, 25 vol % MeOH was chosen to perform electrosyntheses.

**Figure 5:**
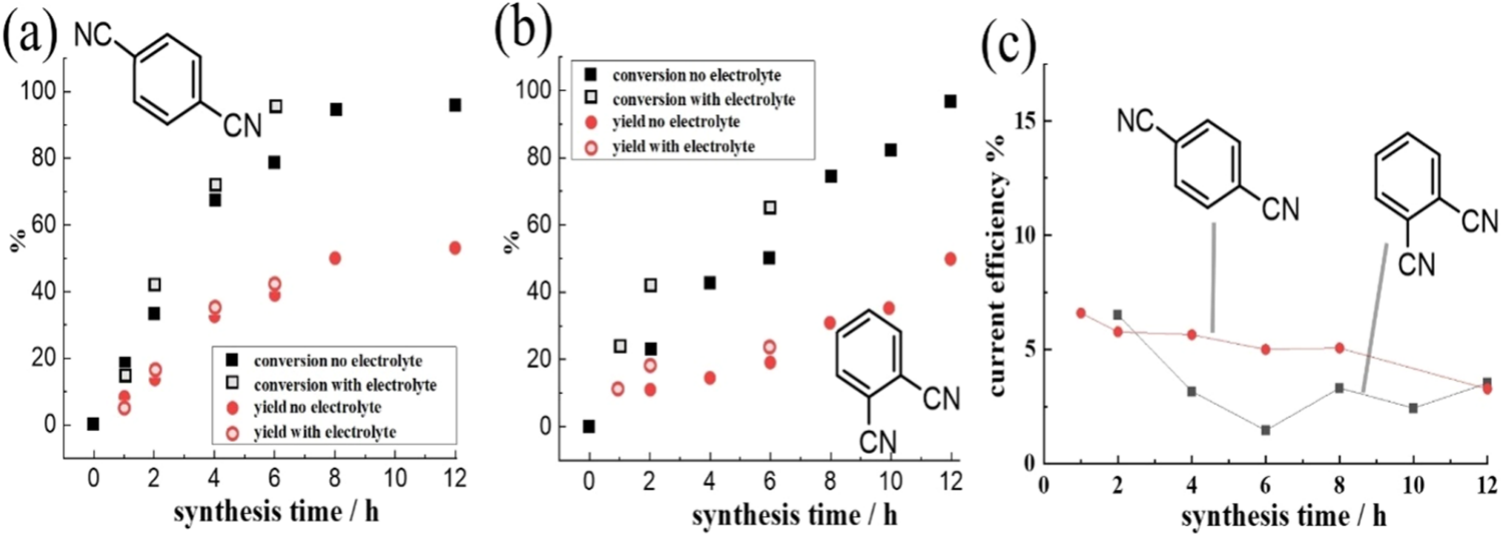
Reaction yield and conversion over time with and without electrolyte 0.1 M TBAP; with 25 vol % methanol; optimized conditions for (a) 0.2 M 1,4-dcyanobenzene and (b) 0.2 M 1,2-dicyanobenzene. The yield was calculated from ^1^H NMR data with mesitylene as internal standard. (c) Current efficiency under optimized conditions using 0.2 M dicyanobenzenes for the synthesis of 4-cyano-benzyl alcohol and 2-cyano-benzyl alcohol.

Figure 5 shows dicyanobenzene electrosynthesis data with different substitution patterns on the aromatic ring. Both, 1,4-dicyanobenzene (Figure 5a) and 1,2-dicyanobenzene (Figure 5b) reach over 95% conversion with about 50% yield at the limit. However, 1,3-dicyanobenzene failed to give any product (not shown; only benzonitrile was identified as product). The resonance effect from the functional groups in 1,4- or in 1,2-position of the aromatic is crucial for determining the reaction pathway (allowing sufficiently stable reactive intermediates to form).

Next, the synthesis conditions with and without added supporting electrolyte were compared (Figure 5). The absence/presence of the supporting electrolyte at the interdigitated microband array electrode has no significant effect on both dicyanobenzene conversion and product yield.

The yield for both 1,4-dicyanobenzene electrolysis and 1,2-dicyanobenzene electrolysis seems limited to approximately 50%. Therefore, 50% of the starting material was consumed in another process. It might be argued that the production of formaldehyde at the anode occurs with one molecule per every 2 equiv of dicyanobenzene being reduced. This imbalance could lead to side reactions and loss of starting material (*e.g*., making oligomeric products).

The paired electrosynthesis at microband array electrodes can be performed with higher concentrations of up to 1 M dicyanobenzene. However, under these conditions, the current does not scale with substrate concentration (factors such as conductivity in lithographically produced platinum films start playing a role) and the electrosynthesis time is considerably extended. It is interesting to explore the overall current efficiency (the fraction of the current that leads to the product) as a function of synthesis time. Figure 5c suggests that for both 1,4-dicyanobenzene and 1,2-dicyanobenzene, the current efficiency is relatively low at about 5% (slightly decreasing with time). Therefore, redox cycling of a stable intermediate (*e.g*., the radical monoanion) seems likely to consume approximately 95% of the electricity in these processes.

### 3.2 Isotope Effects in Paired Electrosynthesis

**Figure 6:**
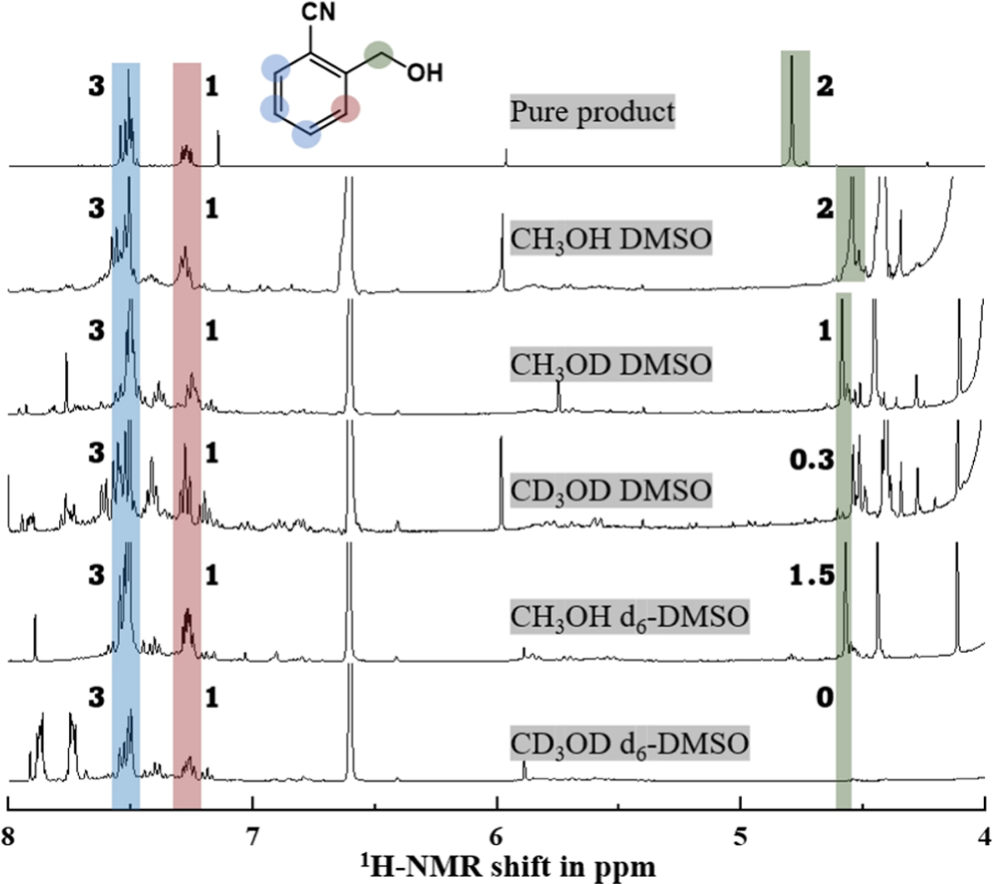
^1^H NMR data showing electrosynthesis products (0.2 M starting material; 4 h synthesis at 4 V; integrals indicated as numbers): (i) pure 2-cyanobenzene methanol reference spectrum in pure CDCl_3_; (ii) product obtained in 25% CH_3_OH in DMSO mixed with 20% CDCl_3_; (iii) product obtained in 25% CH_3_OD in DMSO mixed with 20% CDCl_3_; (iv) product obtained in 25% CD_3_OD in DMSO mixed with 20% CDCl_3_; (v) product obtained in 25% CH_3_OH in *d*_6_-DMSO mixed with 20% CDCl_3_; (vi) product obtained in 25% CD_3_OD in *d*_6_-DMSO mixed with 20% CDCl_3_.

To further explore the mechanism of these reactions, different combinations of deuterated methanol and *d*_6_-DMSO were used to follow the assembly of the product molecule. Figure 6 shows ^1^H NMR data for six different cases. The isolated product in CDCl_3_ shows typical proton NMR signatures for the aromatic protons (blue and red), for the methylene protons (green), and for the hydroxyl proton (broad and solvent sensitive, not indicated). The methylene protons (green) are most diagnostic and shifted to a slightly lower chemical shift in the presence of DMSO. Note: not all peaks are identified/explained, and further complexity might be linked to the formation of unwanted side products (*vide supra*).

When using CH_3_OD in DMSO, a significant change in the ^1^H NMR occurs only for the methylene protons (green; the integrals for aromatic protons blue and red remain, and the integral for methylene in green halves). The deuterium from the hydroxyl group is acidic, and during methanol oxidation, a combination of H^+^ and D^+^ solution species is expected to form. These protons can then affect the product formation (*vide infra*). With CD_3_OH, the signals for the methylene protons (green) are suppressed, indicative of full deuteration. Therefore, the formation of a D_2_C=O intermediate at the anode could be a step in the formation of the methylene group in the product. However, there is a residual signal (integral 0.3) which could indicate a hydrogen atom abstraction from DMSO. This would then lead to a hypothesis of competing H atom abstraction from CD_3_OH and a C-centered radical. When a combination of CD_3_OD and *d*_6_-DMSO, full deuteration of the methylene group is observed.

**Figure 7:**
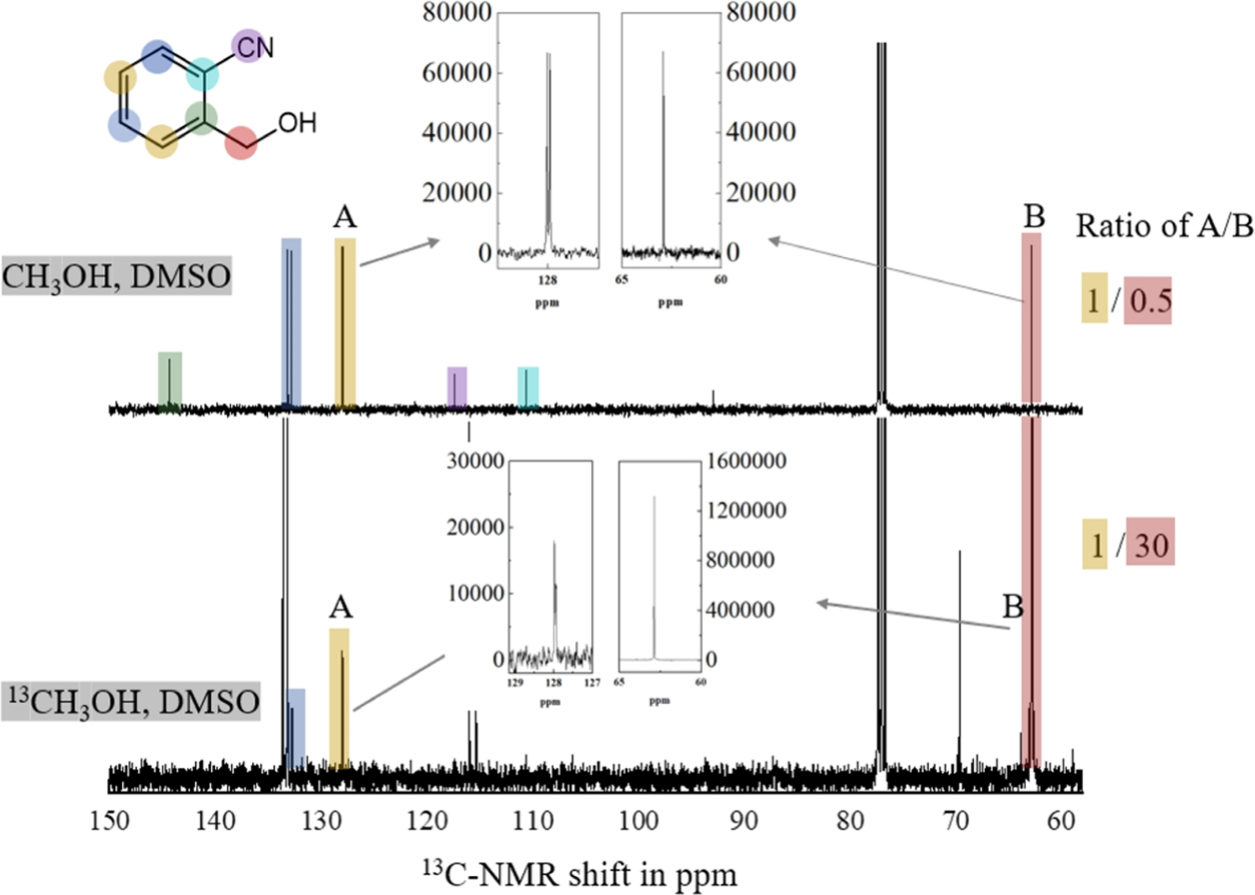
^13^C{^1^H} NMR in CDCl_3_ of cyanobenzyl alcohol products obtained in reactions in the presence of isotopes (^13^CH_3_OH). The integrated intensity of the methylene carbon with ^13^C is 60 times increased.

Next, further experiments were performed to trace the origin of carbon in the methylene group of the product. The electrosynthesis was performed in the presence of ^13^CH_3_OH followed by product analysis with ^13^C{^1^H} NMR. The result in Figure 7 shows that the carbon signal in the methylene (in Ar–CH_2_OH) is approximately 60 times more intense consistent with the incorporation of ^13^C at the methylene group.

**Figure 8:**
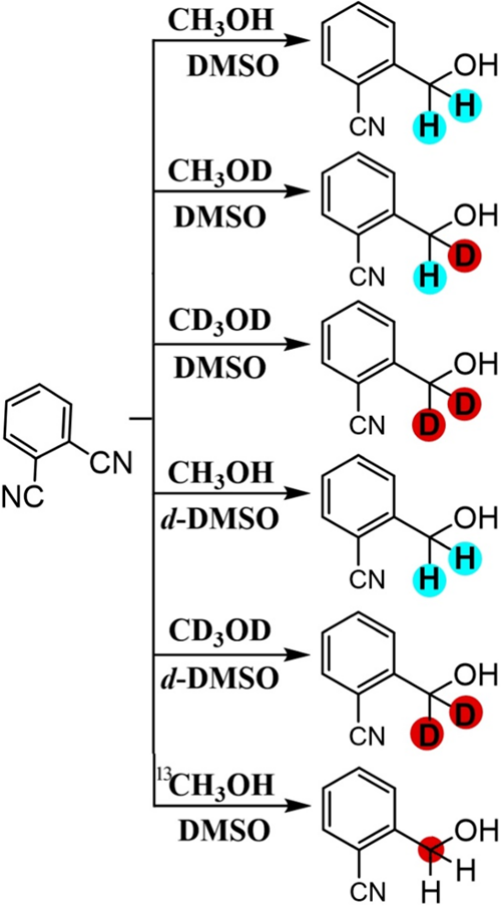
Schematic summary (oversimplified) of isotope effects in the paired electrosynthesis of 1,2-dicyanobenzene.

Figure 8 provides a summary of the isotope effects. Additional experiments exploring the effect of the supporting electrolyte on the isotope distribution suggested that there is no obvious change in mechanism with or without the supporting electrolyte. The methylene must originate from the alcohol. Most intriguing is the formation of the mixed H/D product in the presence of CH_3_OD. The mechanism is discussed next.

### 3.3 Mechanism in Paired Cyanobenzyl Alcohol Electrosynthesis

**Scheme 2:**
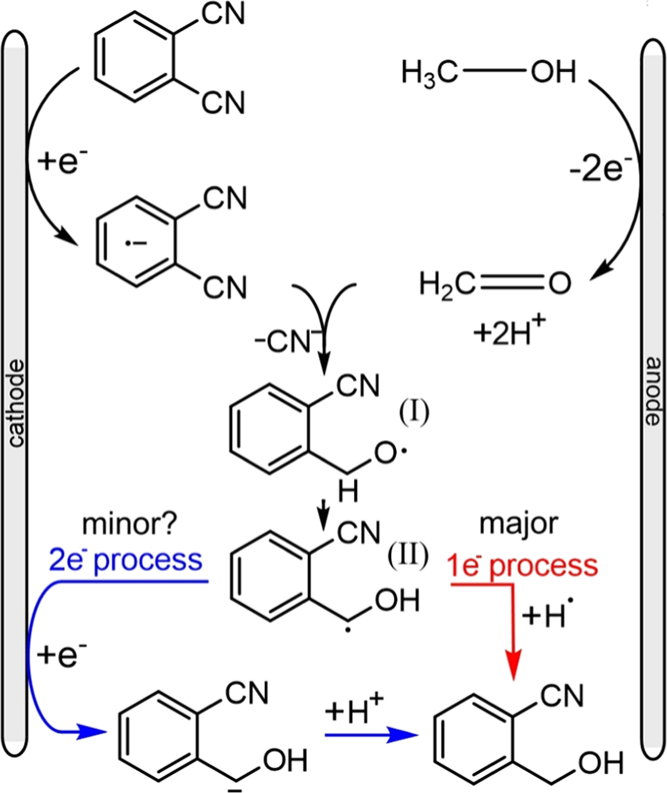
Proposed Mechanism for Paired Electrosynthesis of Cyanobenzyl Alcohol

A proposed mechanism is shown in Scheme 2. Dicyanobenzene is proposed to undergo a one-electron reduction to give an anion radical. Meanwhile, methanol is oxidized at the anode, generating aldehyde in a two-electron process. The anion radical and aldehyde interact in the solution in the diffusion zone to form intermediate **I**. This must transform to intermediate **II**.

To assess the stability of the radical intermediates **I** and **II** (see Scheme 2), density functional theory (DFT) calculations were performed (BP86-D3^BJ^(DMSO)/6-311++G**//BP86/6-311++G**; for further methodology details, see the Supporting Information). For the 1,2-dicyanobenzene structures, the carbon-based radical (**II**) was favored by over 22 kcal mol^–1^, (Δ*G*_DMSO_ = −22.6 kcal mol^–1^) when compared to the oxygen-based radical (**I**). A similar stability was also observed for the equivalent 1,4-dicyanobenzene intermediate species, again with the carbon-based radial structure, *para*-**II**, more stable by Δ*G*_DMSO_ = −24.3 kcal mol^–1^. This strongly implies that carbon radical **II** is preferred thermodynamically and produced *via* a 1,2-shift.

Next, there are two possible pathways. The resulting radical **II** could obtain an electron (either from the cathode or more likely from radical anions), and then react with a H^+^ and subsequently form the final product. In the isotope tracer experiments, when replacing CH_3_OH with CH_3_OD in the synthesis, the product observed from the NMR is monodeuterated. This suggested that there could be further reduction and protonation of the carbanion species. This would result in overall two electrons being consumed at the cathode per dicyanobenzene, thereby balancing the electron count at the anode and cathode. Alternatively, radical intermediate **I** with a radical on the methylene carbon could undergo H atom transfer with either methanol or DMSO (as supported by isotope tracer experiments). As a result, a one-electron process occurs at the cathode, and the overall process for anode and cathode is imbalanced. In the ^1^H NMR data (Figure 6), the methylene protons show an integral of 1.5 (CH_3_OH and *d*_6_-DMSO) and 0.3 (CD_3_OD and DMSO) indicative of H atom transfer. This suggests that the one-electron pathway *via* H atom transfer is the dominant process. However, the data are not conclusive.

The formation of the H/D exchanged product in CH_3_OD could also be linked to isotope exchange for formaldehyde under acidic conditions^32−35^ leading to HDC=O and in this way to the observed products. Therefore, the paired electrolysis process is complex, with many possible intermediates/pathways and with details not fully resolved. In the future, better tools (both experimental and theoretical) will be important to develop in the future.

## 4 Conclusions and Outlook

It has been shown that paired C–C coupling processes at interdigitated microband electrodes are feasible. Although conversion (loss of starting material) is high and current yields reasonable (typically 5%), yields do not progress beyond approximately 50%. This has been attributed to an anode/cathode imbalance in the mechanism, with only one formaldehyde generated for every two dicyanobenzene reduction events. The resulting side products have not been identified.

Electrosynthesis at interdigitated microband electrode arrays allows conditions such as those under mild agitation or in microflow reactors to be utilized without the need for added supporting electrolyte. Ionic species are generated close to anode and cathode (in the interelectrode diffusion zone), and these are sufficient to sustain the electrolysis essentially without Ohmic energy losses. This allows processes to be performed without additional separation and recovery of the electrolyte.

In the future, lithographically produced platinum microband arrays may not be sufficiently robust for long-term operation under industrial electrolysis conditions. New designs and electrode fabrication methods will be important. Microband arrays with a wider range of electrode materials will be desirable. Electrodes directly embedded into reaction ware such as laboratory stirrers will be useful. Future computational approaches combining finite element methods and DFT methods will help further unravel complex mechanisms.

## Supporting Information Available

Computational methodology (Section S1), breakdown of energy contributions (Section S2), and Cartesian coordinates and raw electronic energies (Section S3) (PDF)
