# Supplementary material for: Paired Electrosynthesis at Interdigitated Microband Array Electrodes without Intentionally Added Electrolyte: C–C Coupling of Dicyanobenzenes with Methanol
Source: J Phys Chem C Nanomater Interfaces. 2025 Feb 3;129(6):3014–21. doi: 10.1021/acs.jpcc.4c07899 (PMC11833768; doi:10.1021/acs.jpcc.4c07899)
Supplement: Supplementary file 1 — jp4c07899_si_001.pdf [file jp4c07899_si_001.pdf]

Supporting Information

Paired Electrosynthesis at Interdigitated Microband Array Electrodes without Intentionally Added Electrolyte：C-C Coupling of Dicyanobenzenes with Methanol

Tingran Liu, Claire McMullin, James E. Taylor, and Frank Marken*

Department of Chemistry, University of Bath, Claverton Down, Bath BA2 7AY, UK

Content

1. Computational Methodology 2

2. Breakdown of Energy Contributions 2

Table S1 3

Figure S1 4

3. Cartesian Coordinates and Raw Electronic Energies (in Hartrees) 5

1. Computational Methodology

DFT calculations were run with Gaussian 16 (C.01) with atoms described with the 6-311++G** basis set. Initial BP86 optimizations were performed using the 'grid = ultrafine' option, with all stationary points being fully characterized via analytical frequency calculations as minima (all positive eigenvalues). All energies were recomputed to correct for the effect of DMSO (ε = 46.826) solvent and dispersion. Single point calculations were run using the polarizable continuum model, using the keyword "scrf=DiMethylSulfoxide", and employing Grimme's D3 parameter set with Becke-Johnson damping as implemented in Gaussian.

2. Breakdown of Energy Contributions

The following tables detail the evolution of the relative energies as the successive corrections to the initial SCF energy are included. Terms used are:

ΔE SCF energy computed with the BP86 functional with 6-311++G**

ΔH Enthalpy at 0 K with 6-311++G**

ΔG Free energy at 298.15 K and 1 atm with 6-311++G**

ΔG_{DMSO} Free energy corrected for DMSO solvent and dispersion effects with 6-311++G**

In each case the final data used in the main article are highlighted in bold. Free energies are quoted in kcal mol^{-1}, and include a single point correction for solvation and dispersion.

Table S1. Relative energies for computed doublet radical structures (in kcal mol^{-1}). Data in bold is at the same level as free energies used in the text. Free energies are quoted relative to *ortho*-**I**.

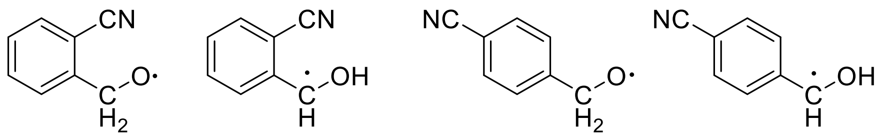

ortho-I *ortho*-II *para*-I *para*-II

	ΔE	ΔH	ΔG	ΔG_{DMSO}
ortho-**I**	0.0	0.0	0.0	**0.0**
ortho-**II**	−20.8	−19.4	−19.2	**−22.6**
para-**I**	0.0	−0.1	−0.3	**−0.8**
para-**II**	−24.4	−23.0	−22.7	**−25.1**

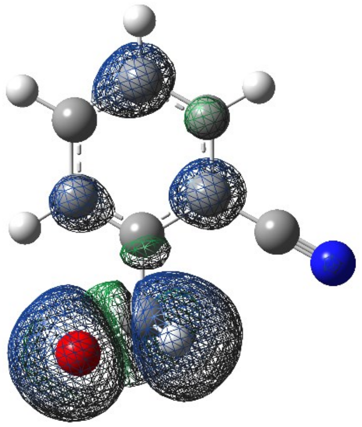

ortho-**I**

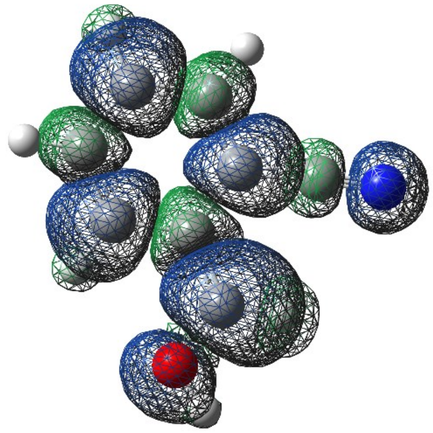

ortho-**II**

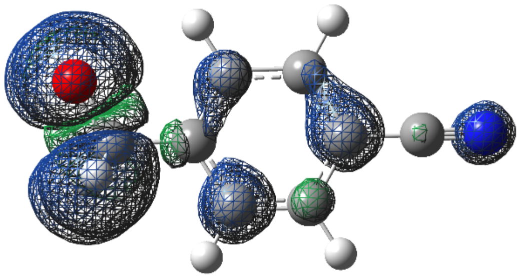

para-**I**

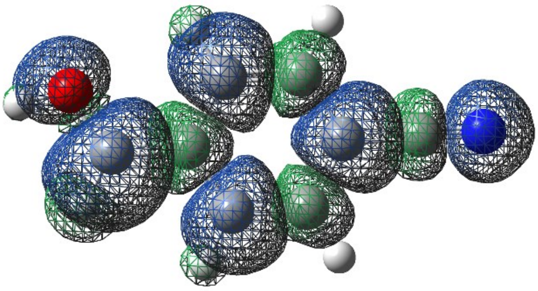

para-**II**

Figure S1. Spin density plots for the radical structures I and II generated with GaussView (at the BP86/6-311++G** level of theory)

3. Cartesian Coordinates and Raw Electronic Energies (in Hartrees)

ortho-**I**
SCF (BP86) Energy = -438.463530504
Enthalpy 0K = -438.351021
Enthalpy 298K = -438.341242
Free Energy 298K = -438.385894
Lowest Frequency = 65.9159 cm^{-1}
Second Frequency = 118.2265 cm^{-1}
SCF (BP86-D3BJ/DMSO) Energy = -438.501541174

C -0.08179 -0.86686 -0.00004
C 0.23951 0.51590 0.00001
C -0.80354 1.45046 0.00003
C -2.13888 1.03130 0.00003
C -2.45288 -0.33714 0.00000
C -1.42922 -1.28723 -0.00002
H -0.54261 2.51163 0.00004
H -2.94061 1.77421 0.00006
H -3.49531 -0.66392 -0.00000
H -1.65589 -2.35544 -0.00006
C 0.96248 -1.84796 -0.00013
N 1.82166 -2.64221 0.00013
C 1.68871 0.97786 -0.00002
H 2.24813 0.50412 0.85493
H 2.24809 0.50415 -0.85501
O 1.93503 2.30033 -0.00000

ortho-**II**
SCF (BP86) Energy = -438.496623881
Enthalpy 0K = -438.381996
Enthalpy 298K = -438.372095
Free Energy 298K = -438.416534
Lowest Frequency = 67.9453 cm^{-1}
Second Frequency = 153.8643 cm^{-1}
SCF (BP86-D3BJ/DMSO) Energy = -438.540011291

C 0.06128 0.66340 -0.00003
C -0.01880 -0.78165 0.00002
C -1.32898 -1.36463 0.00005
C -2.47567 -0.58879 0.00003
C -2.38322 0.82161 -0.00004
C -1.12570 1.42715 -0.00006
H -1.40932 -2.45567 0.00008
H -3.45586 -1.07226 0.00004
H -3.28529 1.43674 -0.00007
H -1.03881 2.51608 -0.00009

C 1.29414 1.38165 0.00003
N 2.25577 2.05104 0.00009
C 1.08162 -1.65596 0.00003
H 0.93092 -2.73909 0.00023
O 2.35724 -1.19059 -0.00010
H 2.98203 -1.93514 -0.00019

para-**I**
SCF (BP86) Energy = -438.463583999
Enthalpy 0K = -438.351239
Enthalpy 298K = -438.341403
Free Energy 298K = -438.386304
Lowest Frequency = 54.9258 cm^{-1}
Second Frequency = 92.0845 cm^{-1}
SCF (BP86-D3BJ/DMSO) Energy = -438.502529824

C -1.52305 -0.01965 -0.00004
C -0.70603 -1.17345 0.00019
C 0.68311 -1.04324 0.00021
C 1.27798 0.22876 0.00012
C 0.46188 1.37619 -0.00006
C -0.92879 1.26129 -0.00018
H -1.17210 -2.16103 0.00042
H 1.32850 -1.92494 0.00005
H 0.91827 2.37156 -0.00020
H -1.56266 2.15027 -0.00050
C -2.94838 -0.14862 -0.00006
N -4.11334 -0.25347 -0.00006
C 2.79062 0.37468 0.00053
H 3.10788 1.03058 0.86046
H 3.10827 1.03286 -0.85766
O 3.55264 -0.73260 -0.00079

para-**II**
SCF (BP86) Energy = -438.502378536
Enthalpy 0K = -438.387610
Enthalpy 298K = -438.377731
Free Energy 298K = -438.422113
Lowest Frequency = 74.5500 cm^{-1}
Second Frequency = 131.7615 cm^{-1}
SCF (BP86-D3BJ/DMSO) Energy = -438.544153184

C 1.53698 -0.02288 0.00001
C 0.69907 -1.17047 0.00008
C -0.67933 -1.04005 0.00011
C -1.29160 0.25638 0.00008
C -0.43434 1.40511 -0.00002
C 0.94210 1.26901 -0.00004
H 1.15674 -2.16225 0.00008
H -1.31421 -1.92772 0.00014

H	-0.87902	2.40447	-0.00006
H	1.58409	2.15238	-0.00012
C	2.95332	-0.16365	-0.00003
N	4.12002	-0.27874	-0.00004
C	-2.68534	0.42651	0.00029
H	-3.15148	1.41542	-0.00038
O	-3.50599	-0.66453	-0.00052
H	-4.43344	-0.37469	0.00193
